# The acute cross-education effect of foam rolling on the thigh muscles in patients after total knee arthroplasty

**DOI:** 10.3389/fresc.2024.1433231

**Published:** 2024-10-25

**Authors:** Masanobu Yokochi, Masatoshi Nakamura, Ayaka Iwata, Ryota Kaneko, Noboru Yamada, Andreas Konrad

**Affiliations:** ^1^ Department of Rehabilitation, Takeda General Hospital, Aizuwakamatsu, Japan; ^2^ Department of Rehabilitation, Fukushima Medical University, Fukushima, Japan; ^3^ Faculty of Rehabilitation Sciences, Nishi Kyushu University, Kanzaki, Japan; ^4^ Department of Orthopaedic Surgery, Takeda General Hospital, Aizuwakamatsu, Japan; ^5^ Institute of Human Movement Science, Sport and Health, University of Graz, Graz, Austria

**Keywords:** pain, range of motion, stiffness, collateral effect, knee osteoarthritis

## Abstract

**Introduction:**

In the early postoperative period after total knee arthroplasty (TKA), joint range of motion (ROM) limitation and increased stiffness due to pain are commonly observed. Previous studies have reported that a single bout of foam rolling (FR) can acutely increase ROM and pain threshold on the contralateral (non-intervention) side in healthy participants. In this study, we aimed to expand this knowledge for TKA rehabilitation and investigated the acute effects of FR intervention on the non-operative side on ROM, stiffness, and pain of the operative side in postoperative patients within the first week after TKA.

**Materials and methods:**

The study employed a randomized crossover design: 20 patients (mean age 75.0 ± 7.8 years) in the first postoperative week after TKA were divided alternately into Roll_Break and Break_Roll groups in the order of prescription. In the Roll_Break group, after the initial evaluation, a 180-s (60-s × three sets) FR intervention using a roller massager by a physiotherapist for the knee extensors was performed on the contralateral side (non-operative side), followed by the measurement. Afterwards, after 180-s of supine at rest, the measurement was performed again (i.e., control phase). In the Break_Roll group, after the initial evaluation, each patient was placed in a seated resting position for 180-s, and then another measurement was performed (i.e., control phase). After this, the FR intervention was performed for 180-s, and then the measurement was performed again. The intensity of the FR intervention was set to the maximum intensity that did not cause pain. We measured pain using the visual analogue scale at rest and during the knee joint ROM measurements, knee joint active movement ROM, knee joint passive ROM, and stiffness during the knee joint active movement.

**Results:**

All outcome variables showed significant improvements after the FR intervention (intervention phase) when compared pre- to post-intervention, and significantly favourable effects were found compared to the control condition.

**Conclusion:**

The results showed significant improvements in ROM, pain, and stiffness of the operative side after the FR intervention on the non-operative side. For future therapy approaches for TKA patients, FR treatment of the non-operative side should be employed in the first weeks after surgery.

## 1 Introduction

Total knee arthroplasty (TKA) can reduce pain and improve knee joint range of motion (ROM) in patients with end-stage knee osteoarthritis. However, clinically, there have been cases of residual pain and limited knee joint ROM due to stiffness after TKA. Previous studies have identified acute postoperative pain as a risk factor for chronic pain after arthroplasty. In addition, knee joint ROM limitation due to stiffness may persist after TKA (1). In previous studies, the incidence of stiffness has been reported to range between 8% and 12% (2–4). It has been pointed out that limited knee flexion ROM due to increased stiffness after TKA may interfere with patients’ activities of daily living (ADL) and cause increased chronic pain and decreased knee functional scores (5). Thus, pain in the early postoperative period after TKA is associated with decreased knee joint ROM and the development of chronic pain. Therefore, early postoperative pain management is important.

In sports, foam rolling (FR) has attracted attention as a method to alleviate pain and improve joint ROM without involving joint movement, serving as an alternative to stretching interventions (6, 7). We have previously reported that FR intervention using a roller massager on the quadriceps muscle in the second week after TKA surgery resulted in an immediate reduction in pain during knee flexion and an increase in joint ROM (8). In addition, a 6-min daily FR intervention on the quadriceps muscle for 1 week from the second to the third week after TKA surgery, led to a significant decrease in pain during knee flexion (9). Thus, FR intervention may be useful for treating pain and decreased joint ROM in postoperative TKA patients.

On the other hand, walking and ADL exercises are often prescribed in the first postoperative week, requiring earlier pain management and improved knee joint ROM. However, introducing FR interventions as early as the first postoperative week may cause pain and discomfort around the surgical wound and should be carefully considered. Interestingly, previous studies have shown that a unilateral FR intervention can increase the pain threshold and improve ROM on the contralateral (non-intervention) side (10–13). This phenomenon is called the “cross-education (contralateral) effect.” In a previous study (12), we investigated the cross-education effect of a vibration FR intervention on ROM, muscle soreness, and pressure pain threshold in eccentrically damaged muscles. The results showed that the vibration FR intervention on the non-damaged side significantly improved the damaged muscle in knee flexion ROM, reduced muscle soreness at palpation, and increased pain threshold (12). The cross-education effect may be explained by the activation of the descending anti-nociceptive pathway [diffuse noxious inhibitory control (DNIC)] (14, 15). It is elicited by nociceptive stimuli (i.e., heat, high pressure, electrical stimulation) and transmits signals from the spinal cord to the brain. As a result, the brain monoaminergically inhibits pain transmission, reducing pain perception locally and at distant sites (16). Therefore, performing an FR intervention on the non-operative side in TKA patients during the first postoperative week may improve the pain and functional decline on the operative side. Therefore, this study aimed to clarify whether a single FR intervention on the non-operative lower limb can effectively improve the pain and ROM of the operative lower limb of the participants in the first postoperative week after TKA. Based on the previous study (12), we hypothesized that an FR intervention on the non-operative side would improve the pain and functional decline on the operative side in TKA patients.

## 2 Methods

### 2.1 Experimental setup

**Figure 1:**
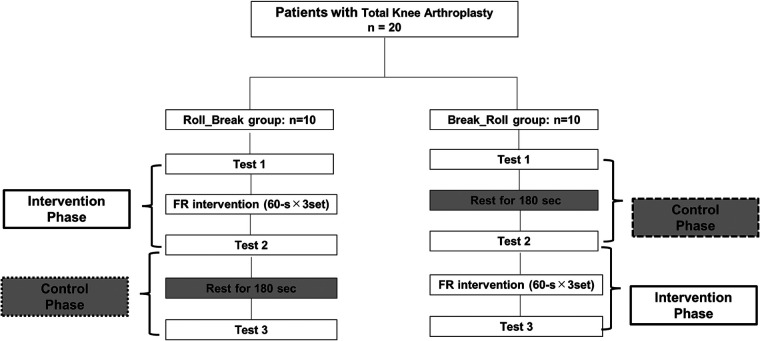
Experimental flowchart.

The study was a randomized crossover study to investigate whether an FR intervention on the non-operative lower limb of postoperative TKA patients has an acute effect on pain, stiffness, and knee ROM in the operative lower limb. Since the measurements in this study were performed in the afternoon of the first postoperative week, there was a possibility that the timing of the measurements could have affected the results. Therefore, we defined the intervention phase as the change before and after the FR intervention. In addition, the control phase was defined as the change before and after the FR intervention while the patients were in a resting condition for the same duration as the FR intervention. The experimental workflow is shown in Figure 1. In total, 20 TKA patients were assigned alternately to either the Roll_Break or Break_Roll group according to the prescription order. In the Roll_Break group, after the initial evaluation (Test 1), a 180-s FR intervention was performed, followed by the measurements (Test 2). Afterward, after 180 s of rest in a supine position, the measurements were performed again (Test 3). In the Break_Roll group, after the initial evaluation (Test 1), each patient rested in a supine position for 180 s, and then the measurements were performed (Test 2). After this, the FR intervention was performed for 180 s, and then the measurements were performed again (Test 3). The 180-s rest period was implemented to check the carryover effect from the joint ROM measurements and the FR intervention. We measured pain, knee joint active ROM, knee joint passive ROM, and stiffness during the knee joint active movement.

### 2.2 Participants

The participants were 20 patients admitted to the hospital who had undergone TKA for knee osteoarthritis between March 2023 and September 2023. Inclusion criteria included patients with no intraoperative problems who could practice joint ROM and gait exercises from the day after surgery. Exclusion criteria included patients who could not consent to the study, those undergoing revision TKA, and those with a current medical history of rheumatoid arthritis or severe leg paralysis. All patients were informed about the study and provided informed consent during the first postoperative week after TKA. The mean age of the FR group participants was 75.0 ± 7.8 years (range: 59–90 years), with 75.0% (*n* = 15) being women. Nine of the 20 patients had surgery on the right knee joint. None of the patients experienced serious complications related to motor function, and no postoperative complications were observed. Preoperative motor function was independent walking in all patients. Medical history included high blood pressure in two patients, type 2 diabetes in six, deep vein thrombosis in seven, osteoporosis in two, and angina pectoris in one. The surgical technique used was a medial parapatellar approach with a cruciate-substituting implant (Zimmer Biomet, Warsaw, IN, USA). The same doctor performed all surgeries. All patients were taking pain medication internally and using icing as part of their physical therapy regimen. Ethical review was carried out after approval by the Takeda General Hospital's Ethical Review Committee (approval number: R4-029). The study was registered with the University Hospital Medical Information Network Clinical Trials Registry (UMIN000055476).

### 2.3 Physical therapy intervention

The physical therapy intervention included wheelchair use, joint mobilization exercises, strength training, and walking exercises with walking aids for 40 min twice daily, once in the morning and once in the afternoon, for a total of 80 min/day, six times per week, according to pain level and the general condition of the patient from the day after surgery, under the direction of the doctor.

### 2.4 Foam rolling exercise

**Figure 2:**
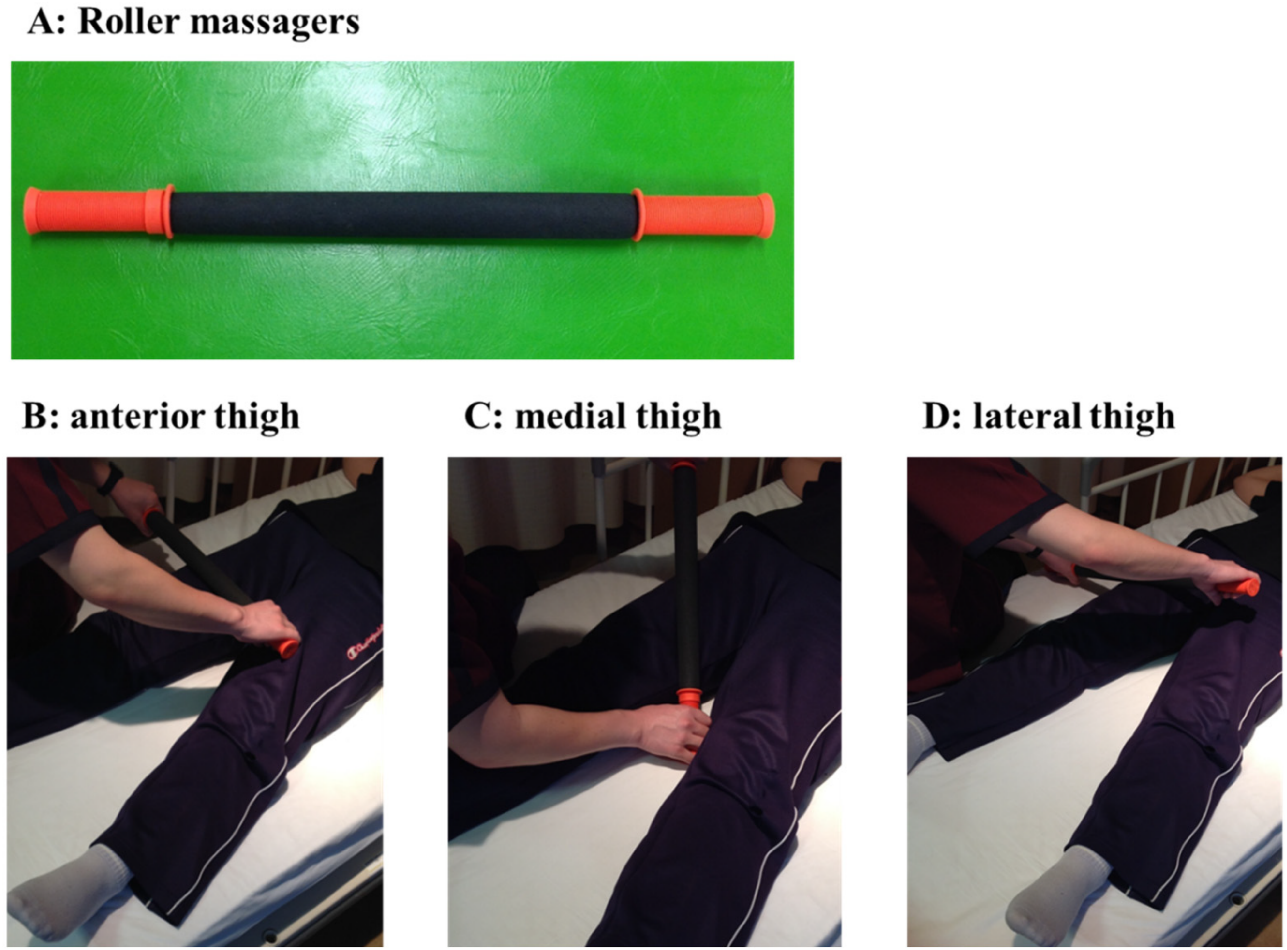
A roller massager and foam rolling intervention methods used in this study.

A roller massager (Tiger Tail, Kent, WA, USA) was applied to the non-operative lower limb by a physiotherapist in the first postoperative week (Figure 2A). The FR intervention targeted sites from the suprapatellar border to the hip, anterior thigh, medial thigh, and lateral thigh, considering previous studies (8, 9, 17) (Figures 2B–D). Each FR intervention was performed for 60 s. The intensity of the FR intervention was set to the maximum intensity that did not cause pain. The posture was supine on the bed with the knee joint in extension. The patient remained in the supine position on the bed and relaxed during the 180 s.

### 2.5 Knee flexion and extension ROM measurement

A physiotherapist with at least 10 years of experience used a goniometer to measure the active and passive knee flexion and extension ROM in increments of 5°. The posture was supine on the bed. The final area of measurement was defined as the point of pain onset. In our previous study, the knee flexion and extension ROM measurement in TKA patients demonstrated high test–retest reliability [intra-class correlation coefficient (ICC) = 0.92 and 1.00, respectively] (8).

### 2.6 Pain measurement

The degree of pain was measured using the Visual Analog Scale (VAS) at rest and during the knee joint ROM measurements. The VAS allows patients to rate their knee pain by marking an X on a 100-mm line (0 mm for no pain, 100 mm for worst possible pain). The VAS has high test–retest reliability (ICC = 0.94) and has been shown to correlate with other tests of pain intensity (18).

### 2.7 Measurement of subjective joint stiffness

The degree of subjective joint stiffness during knee joint active flexion and extension was investigated using a scale similar to the VAS (19). The criteria were 0 mm as no subjective stiffness and 100 mm as the worst subjective stiffness.

### 2.8 Statistical analysis

Statistical analysis was performed using SPSS (version 28.0; IBM Corp., Armonk, NY, USA). The Shapiro–Wilk test was used to assess the normality of the data. We confirmed that the variables followed normality. We compared the baseline measurements (Test 1) between the Roll_Break and Break_Roll groups using an unpaired *t*-test. In the Roll_Break and Break_Roll groups, the change before and after the 180-s FR intervention was defined as the “intervention phase,” and the change before and after the 180-s rest was defined as the “control phase.” We compared all the variables between the intervention and control phases using a paired *t*-test. In addition, we also used a paired *t*-test to compare all the variables before and immediately after the FR intervention.

## 3 Results

**Figure 3:**
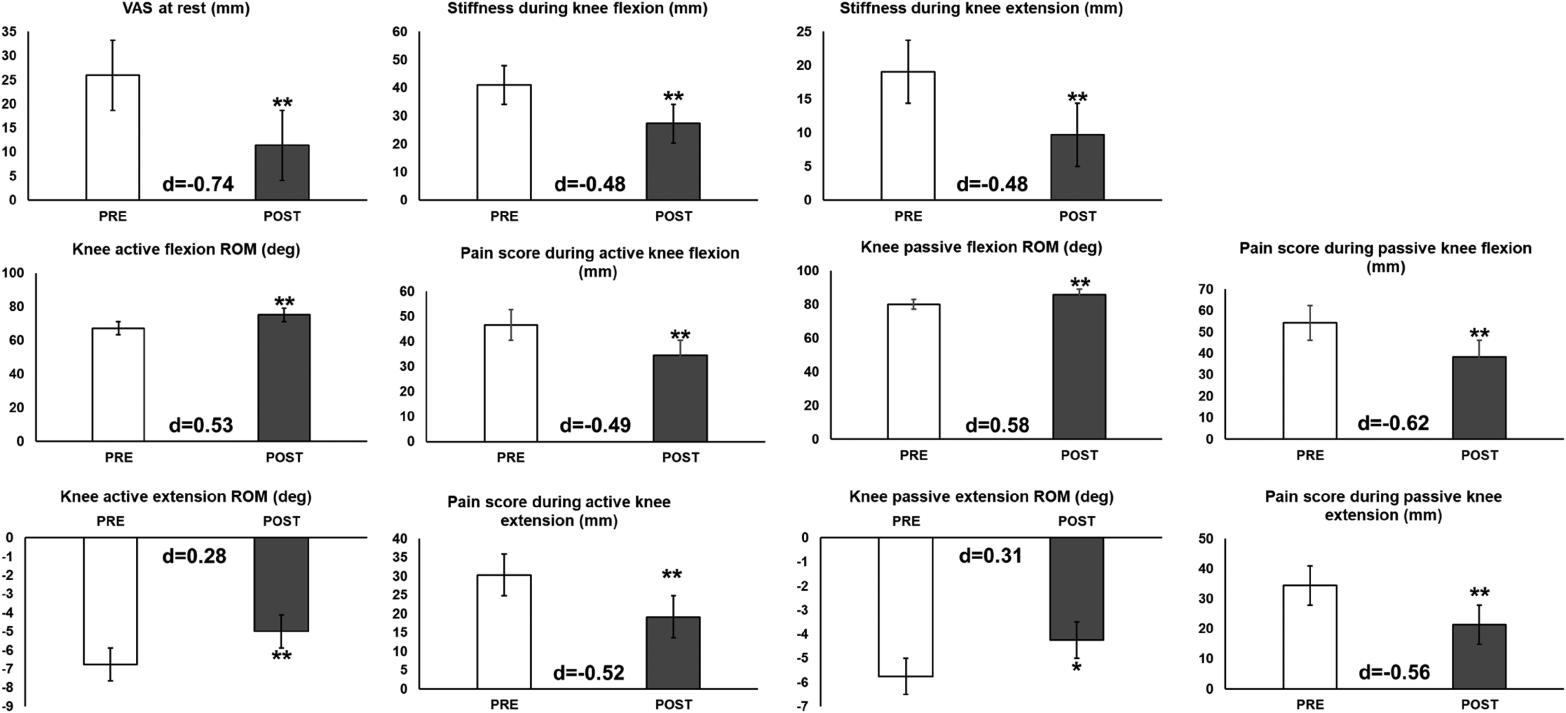
Outcome variables before (PRE) and immediately after the foam rolling intervention (POST) in the intervention phase (*n* = 20). *p < 0.05; **p < 0.01. Significance was tested using paired *t*-tests.

Table 1 lists all the variables in the baseline measurements in both Roll_Break and Break_Roll groups. Pain scores during active knee extension and stiffness during knee extension in the Break_Roll group were significantly higher than in the Roll_Break group, but there were no significant differences between the groups for the other variables. Table 2 lists the changes in all variables in the intervention and control phases. A paired *t*-test shows significant improvements in all variables in the intervention phase compared to the control phase. The changes in all variables before and immediately after the FR intervention are shown in Figure 3. A paired *t*-test shows a significant (*p* < 0.05) improvement in all variables immediately after the FR intervention.

**Table 1:** Changes in each variable (mean ± SD) in the intervention and control phases.

Baseline measurement	Roll_Break group, *N* = 10	Break_Roll group, *N* = 10	Unpaired *t*-test
Pain score at rest (mm)	22.5 ± 21.7	29.4 ± 24.6	*p* = 0.54
Knee joint active flexion ROM (°)	70.0 ± 15.5	64.5 ± 14.6	*p* = 0.45
Pain score during active knee flexion (mm)	35.7 ± 20.8	55.7 ± 24.3	*p* = 0.054
Knee joint passive flexion ROM (°)	81.5 ± 11.6	78.5 ± 10.5	*p* = 0.57
Pain score during active knee motion (mm)	44.0 ± 20.1	66.4 ± 22.8	*p* = 0.06
Stiffness during knee flexion (mm)	29.0 ± 20.8	53.1 ± 33.1	*p* = 0.08
Knee joint active extension ROM (°)	−7.0 ± 7.1	−6.5 ± 5.0	*p* = 0.87
Pain score during active knee extension (mm)	19.0 ± 12.2	41.7 ± 24.3	*p* = 0.02
Knee joint passive extension ROM (°)	−6.0 ± 5.4	−3.5 ± 6.3	*p* = 0.38
Pain score during passive knee extension (mm)	26.1 ± 19.3	42.7 ± 25.6	*p* = 0.14
Stiffness during knee extension (mm)	8.0 ± 12.5	30.1 ± 27.0	*p* = 0.04

**Table 2:** Changes in each variable (mean ± SD) in the intervention and control phases.

	Roll_Break group (*n* = 10)		Break_Roll group (*n* = 10)		All participants (*n* = 20)	
	Intervention phase	Control phase	Intervention Phase	Control phase	Intervention phase	Control phase
Pain score at rest (mm)	17.2 ± 21.9	2.0 ± 6.0	11.9 ± 8.9	0	14.6 ± 17.1 *p* = 0.005	1.0 ± 4.4* d = 1.27
Knee joint active flexion ROM (°)	−8.5 ± 9.0	0	−7.5 ± 3.8	0	−8.0 ± 7.0 *p* = 0.000	0* d = −2.30
Pain score during active knee flexion (mm)	11.0 ± 13.4	1.0 ± 3.0	13.3 ± 9.4	0	12.2 ± 11.8 *p* = 0.001	0.5 ± 2.2* d = 1.66
Knee joint passive flexion ROM (°)	−7.0 ± 5.6	0	−5.0 ± 3.7	0	−6.0 ± 4.9 *p* = 0.000	0* d = −2.45
Pain score during passive knee flexion (mm)	16.6 ± 16.9	6.0 ± 18	15.5 ± 10.0	0	16.1 ± 14.1 *p* = 0.016	3.0 ± 13.1* d = 0.96
Stiffness during passive knee flexion (mm)	5.9 ± 8.7	3.5 ± 9.0	21.6 ± 14.1	0	13.8 ± 14.5 *p* = 0.007	1.75 ± 6.6* d = 1.14
Knee joint active extension ROM (°)	−1.5 ± 2.3	−0.5 ± 1.5	−2.0 ± 2.3	3.0 ± 5.7	−1.8 ± 2.4 *p* = 0.049	1.3 ± 4.7** d = −0.85
Pain score during active knee extension (mm)	9.8 ± 13.2	0.5 ± 1.5	12.5 ± 9.6	0.1 ± 0.3	11.2 ± 11.8 *p* = 0.001	0.3 ± 1.1* d = 1.68
Knee joint passive extension ROM (°)	−2.0 ± 2.4	0	−1.0 ± 1.9	0	−1.5 ± 2.3 *p* = 0.010	0** d = −1.31
Pain score during passive knee extension (mm)	12.6 ± 13.7	0.2 ± 0.6	13.6 ± 9.8	0	13.1 ± 12.1 *p* = 0.000	0.1 ± 0.4* d = 2.08
Stiffness during passive knee extension (mm)	3.0 ± 6.4	1.9 ± 5.7	15.7 ± 10.8	0	9.4 ± 11.2 *p* = 0.009	0.95 ± 4.1* d = 1.10

* *p* < 0.01; **p < 0.05.

## 4 Discussion

This study investigated the effects of an FR intervention on a non-operative lower limb on pain, knee joint ROM, and stiffness on the operative side, i.e., the cross-education effect, in TKA patients during the first postoperative week. The results showed that the FR intervention on the non-operative side significantly improved all variables on the operative side. To the best of our knowledge, this is the first study to investigate the cross-education effect of FR intervention in postoperative TKA patients. A systematic review has shown the effectiveness of rehabilitation and outpatient therapy in the early postoperative period after TKA surgery (20). FR intervention in the non-operative lower extremity can be performed early in the postoperative period and can be applied as independent training after discharge, which may contribute to increasing the effectiveness of rehabilitation.

In this study, pain at rest and during knee flexion and extension significantly improved immediately after the FR intervention. Nakamura et al. (12) investigated the cross-education effect of a vibration FR intervention on ROM, muscle soreness, and pain threshold in eccentrically damaged muscles of young, healthy, sedentary participants. They showed that the vibration FR intervention on the contralateral side can improve ROM and reduce muscle soreness in eccentrically damaged muscles. The results are similar to those of the previous study and ours, although the present study differs in that the participants were TKA patients. The previous study by Aboodarda et al. (21) suggested that FR interventions may reduce pain sensation by activating the ascending pain inhibitory system, the descending anti-nociceptive pathway, and the autonomic nervous system. While the details of the mechanism by which pain was improved in the postoperative TKA patients in this study remain unclear, we suspect that a similar mechanism may have achieved the cross-education effect. The FR intervention on the non-operative side reduced pain during quadriceps muscle extension and contraction, which may have been the reason for the improved active and passive knee flexion and extension ROM.

The FR intervention on the non-operative lower limb also improved stiffness during knee flexion and extension, which has been reported to occur in 4%–16% of postoperative patients after TKA surgery and is an important factor contributing to pain and functional limitations (22). In addition, a previous study described the importance of preventing stiffness through pain-free physiotherapy after TKA (23). Zaffagnini et al. (24) reported that pain and anxiety were associated with postoperative stiffness after TKA. In this study, the FR intervention on the non-operative lower limb provided pain relief, as described above. This may have reduced pain and fear of joint movement, leading to decreased stiffness.

TKA has been reported to cause significant acute postoperative pain despite analgesics (1). In addition, aggressive joint mobilization exercises after TKA may lead to wound dehiscence. Prolonged drainage due to wound dehiscence has been reported to increase the risk of infection (25). On the other hand, the FR intervention on the non-operative lower limb in this study did not directly intervene on the operative side, so there was no risk of wound dehiscence, indicating that it may be possible to manage pain safely and improve joint ROM. Kocic et al. stated that fear of movement after TKA surgery is associated with knee flexion ROM (26). Bakırhan et al. also reported that increased pain and decreased quadriceps muscle strength after TKA were associated with fear of exercise (27). For these reasons, we believe that knee joint ROM and pain in the early postoperative period after TKA surgery may lead to fear of exercise. Therefore, employing FR intervention on the non-operative lower extremity to reduce pain in the operative lower extremity is a clinically useful approach. We have previously verified the acute effect of FR intervention on the operative side in the second week after TKA (8). In the previous study, the VAS change during active knee joint flexion was −11.9 ± 21.0 mm (*d* = −0.53), whereas it was −16.1 ± 3.8 mm (*d* = −0.62) in the present study. The change in passive knee flexion ROM was 4.1 ± 3.2° (*d* = 0.412) in the previous study and 6.0 ± 1.8° (*d* = 0.582) in the present study. The change in ROM during knee joint extension was 0.2 ± 1.0° (*d* = 0.055) in the previous study and 1.5 ± 0.6° (*d* = 0.31) in the present study. Although the timing of the intervention was the first postoperative week in the present study compared to the second postoperative week in the previous study, the results indicate that the FR intervention on the non-operative lower limb may have the same effect as that observed in the previous study, in which the intervention was performed directly on the operative side. Thomazeau et al. stated that one factor for chronic postsurgical pain 6 months after TKA is preoperative walking pain (28). Lo et al. also categorized TKA patients into two groups based on their VAS scores on postoperative day 1/2: minor (VAS < 5) and major (VAS ≥ 5) pain groups. They reported that the group with less pain had a higher knee joint function score 6 months and 2 years after TKA (29). Based on the results of this study, it is possible that FR can be performed on the non-operative lower extremity to reduce pain in the operative lower extremity, and rehabilitation activities such as walking can be performed with reduced pain in the operative lower extremity. As described above, physical exercise and aggressive pain relief in the early postoperative period for TKA patients could be beneficial for knee function and pain management. Thus, FR may be applied as a new rehabilitation and/or self-care tool because it does not burden the wound and reduces pain in the operated lower extremity in the early postoperative period in TKA patients.

A limitation of this study was verifying an acute effect, and it is unknown how long the obtained effect will last. Comparisons with the operative lower limb may also differ in pain levels and the degree of improvement in joint ROM due to the different time periods since surgery. Also, since this study had a small sample size (*N* = 20) and a sex bias (75% women), it is necessary to conduct future studies on a larger scale and investigate the sex differences in the effects of FR intervention. In the future, we plan to explore the effects of FR on the operative leg starting in the first postoperative week, FR on the non-operative leg, and FR intervention on the operative leg beginning in the second postoperative week. In addition, it is necessary to investigate the chronic effect of FR intervention on the non-operative leg. Moreover, it is also necessary to compare the cross-education effect of FR intervention with other rehabilitation interventions, such as stretching and dynamic joint movement.

In conclusion, this study investigated the cross-education effect of FR intervention on pain, knee joint ROM, and stiffness of the operative side in TKA patients in the first postoperative week. The results showed significant improvements in knee joint ROM, pain, and stiffness immediately after the FR intervention, with minimal risk to the operative side.

## Data Availability

The raw data supporting the conclusions of this article will be made available by the authors without undue reservation.
